# ED Revisits Within 72 Hours to a Tertiary Health Care Facility in Dubai: A Descriptive Study

**DOI:** 10.7759/cureus.36807

**Published:** 2023-03-28

**Authors:** Maryam Al Ali, Maitha R Alfalasi, Hamda A Taimour, Aiman M Ahmed, Omar Q Muhammed Noori

**Affiliations:** 1 Emergency Department, Rashid Hospital Trauma Centre, Dubai Academic Health Corporation, Dubai, ARE

**Keywords:** emergency department, dubai, tertiary hospital, 72 hours, unplanned revisit

## Abstract

Unplanned emergency department (ED) revisit is one of the major challenges faced by emergency care facilities and reflects their quality of care. It is an important key performance indicator (KPI) for emergency medical care. Often, inadequate medical care by physicians is claimed to be the main cause of unplanned ED revisits, yet this assumption is not well studied in the literature. Thus, this study aimed to identify the causes of unplanned ED revisits within 72 hours from the initial visit to the emergency department which could help in developing an action plan and improve quality of care and patient safety.

A retrospective study was conducted in Rashid Hospital Trauma Center, from December 2019 to January 2020, using electronic medical records reviewed by two independent investigators. The reasons for the ED revisits were categorized into the following four domains: illness, physician, patient, and system related. A total of 584 revisits were found which accounted for 1.9% of ED attendance from December 2019 to January 2020. Majority of them were male patients, and 63% of the population had a mean age of 33 years. Majority of the ED revisits were due to illness (54%), followed by patient related (20%), physician related (18%), and system related (8%) factors. Most of the patients were discharged on the second visit. The two most common reasons for revisits in the ED department that were seen within the 72 hours were illness related and patient related, followed by physician related. The cause is mainly rooted in suboptimal discharge plans.

## Introduction and background

Based on international research data, the unplanned emergency revisit rates ranged from 0.99% to 5.8% according to the revisit timing of 72 hours [1-9]. A number of studies have been identified that looked into the unplanned emergency department (ED) revisits. For instance, a study by Wu et al. at Kuang Tien General Hospital, Taiwan showed that illness-related causes accounted for the most ED revisits, and the most common illness cause was reported to be abdominal pain for these unplanned revisits [8]. Another study was conducted at Singapore General Hospital by Chan et al. and showed that older aged individuals, male gender, Chinese ethnicity, arrival to the hospital by ambulance, abdominal pain, heart disease diagnosis, triaged as category 2, or viral infection were all significantly associated risk factors for ED revisits within 72 hours [5].

Furthermore, a study conducted by Khan et al. at Aga Khan University Hospital, a tertiary care hospital in Karachi, Pakistan, showed that the leading cause of return visits and admission to ED was due to fever and infectious diseases at 29% and 7% due to misdiagnosis [10].

Some studies were also conducted in the Gulf region. A study was conducted at King Abdulaziz Medical City Hospital, located in Al Riyadh city, Saudi Arabia which analyzed the reasons for emergency department revisit and classified them based on system of presenting illness and identified circulatory and genitourinary systems diseases as the major reasons for ED revisits [11]. While in another prospective study of unscheduled revisits within 72 hours to an adult ED in Al Khor Hospital, State of Qatar, classified these revisits into following four major categories which were: illness related, physician related, system related, and patient related. The study highlighted that the majority of ED revisits were due to illness-related or system-related causes [6].

Unplanned ED revisits are one of the major challenges faced by emergency care facilities and reflect their quality of care. It is an important key performance indicator (KPI) for emergency medical care. Often, inadequate medical care by physicians is claimed to be the main cause of unplanned ED revisits, yet this assumption is not well studied in the literature. Thus, this study aimed to identify the causes of unplanned ED revisits within 72 hours from the initial visit to the ED of Rashid Hospital, a government tertiary care hospital in Dubai, United Arab Emirates (UAE), to execute a root cause analysis for the unplanned revisits to the ED, which could help in developing an action plan and improve quality of care and patient safety.

## Review

Materials and methods

This study was conducted at Rashid Hospital Trauma Centre (RHTC) located in Dubai, UAE. It is a teaching hospital and level 1 trauma center with 762 beds and 68 beds in ED. The records of the hospital showed annual emergency visits of 120,000 patients to the ED of the hospital. Patients in ED were seen from different nationalities and economic standards with different education levels, trauma of all ages, pregnancy trauma, and medical emergencies starting from age above 13 years.

Patients with other emergencies that are out of RHTC scope of service are stabilized and transferred to other hospitals like Dubai Hospital, Latifa Women Hospital, and Al Jalila Children Specialty Hospital for further management by subspecialties including ENT, ophthalmology, urology, nephrology, obstetrics and gynecology (OBGYN), pediatrics, neonatology, hematology, oncology, and endocrinology, which are linked with same electronic medical record system. The triaging system adopted in the study hospital is the Canadian Triage and Acuity Scale (CTAS) which categorizes patients into the following five categories: T1 resuscitation, T2 emergent, T3 urgent, T4 less urgent, and T5 non-urgent.

ED is covered by three shift duties per day that are 9.5 hours with 11 emergency physicians distributed in different areas including shift captain, resuscitation, ambulance triage, rapid assessment zone, and major and minor observation areas. Electronic medical record system “Epic” (Verona, WI: Epic Systems Corporation) was initiated in Dubai Academic Health Corporation (DAHC) in 2017 and this system is used only in government hospitals and primary health centers in Dubai.

The current study was a retrospective chart review, conducted during the period from December 1, 2019, to January 31, 2020. All the patients with unplanned revisits to the ED within the past 72 hours with the same or related complaints were included, considering that the first visit at Rashid Hospital ED and the second visit to the same hospital or the other linked DAHC hospitals. The hospital electronic system creates a report for all ED revisits within 72 hours according to the hospital quality assurance indicator regardless of the type of complaints. Whereas exclusion criteria consisted of the discharged patients against medical advice on first visit, a second revisit of a patient to an ED for a different complaint, discharge by inpatient specialty, and a planned visit of the patient to ED by the emergency team of the hospital.

The research team consisted of two emergency physicians and emergency residents and one emergency consultant. The research hypothesis, the data collection tool, data analysis, and the major themes selected for the revisit reasons were all explained in detail to the research team. In addition, before the start of the data collection, each member of the research team was trained on how to select the most appropriate revisit themes for the patient's unplanned revisits to the ED. After ensuring adequate training and education were provided, the research team members worked independently for data collection and analysis. With the aim that each case was reviewed by two research team members independently. Moreover, in case of discrepancy or inconsistency of category, the final verdict would be reviewed by a third reviewer (emergency consultant) different than the first two reviewers.

As shown in Table 1, in this study the revisits to the ED were divided into four categories by the reviewers including - (1) physician related (when patient management in ED was suboptimal including management related, which indicated that the physician made an error during the course of emergency management assessment, investigation, or treatment; diagnosis related, which indicated that the diagnosis made by the first physician was incorrect as determined by the chart review; disposition related, which indicated that the patient was discharged home while they needed an admission, refer to inpatient service or transfer to other hospitals for further treatment; discharge plan related, which indicated that the discharge plan was inadequate for continuity of care that included discharge medications, discharge instructions or follow-up), (2) patient related (when the revisit was related to patient factors after excluding all other reasons, like no compliance of the patient towards the treatment or no new symptoms in the second visit), (3) system related (revisit to ED that was planned by other medical services or due to unavailability of service), and (4) illness related (patient received the proper investigation and treatment in the ED but the disease progressed or failure of standard treatment occurred as per guidelines of the hospital to improve the symptoms, new symptoms occurred, side effects of indicated treatment or revisit to other facility to continue the care, when service was not available in the study hospital).

**Table 1:** Emergency revisits categories.

Emergency revisit categories	Definitions	Subcategories and examples
1. Illness related	Reason related to disease progression or failure of standard treatment (as per international/hospital guidelines), considering that the patient received the proper investigation and treatment in the emergency department	New symptoms occurred
		Side effects of indicated treatment
		Revisit in other facilities to continue the care (when service is not available in our hospital)
2. Patient related	Reasons related to patient’s factors after excluding all other reasons related to the revisit	Patient is not compliant with initial treatment plan
		No new symptoms develop on the second visit
3. Physician related	Reasons related to ED physician management starting from assessment, investigation, diagnosis, disposition, and discharge plan	Management related: the physician made an error during the course of emergency management (assessment, investigation, or treatment)
		Diagnosis related: the diagnosis made by the first physician was incorrect as determined from the chart review
		Disposition related: patient was discharged home while they needed admission, refer to inpatient service, or transfer to other hospitals for further treatment
		Discharge plan related: discharge plan was inadequate for continuity of care (discharge medications, discharge instructions, or follow-up)
4. System related	Reason related to healthcare system services, when services are not available in certain facilities	Revisit to emergency that was planned by other medical services or unavailability of service: multiple sclerosis patient attending ED for steroids injection

All data were recorded and analyzed using Microsoft Excel 2020. All the variables were shown as frequency and percentages were presented as tables and graphs.

Results

**Figure 1:**
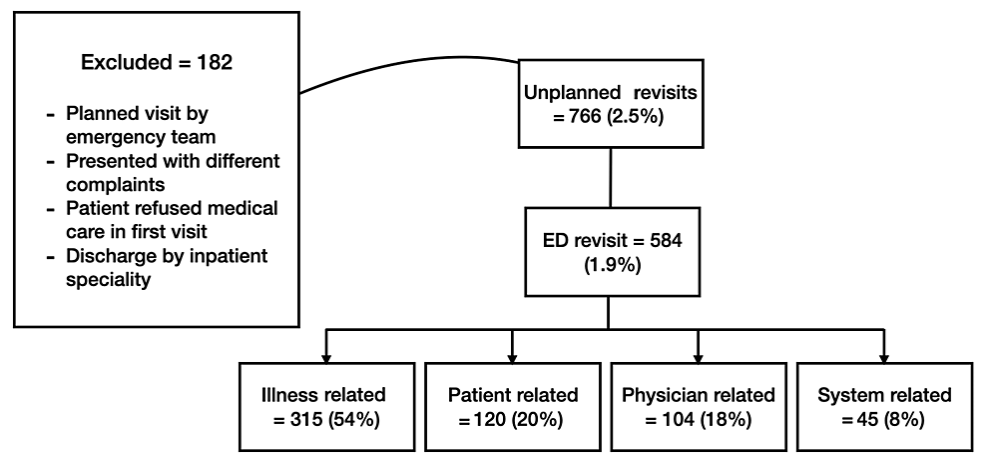
Inclusion and exclusion criteria.

Return visits to the ED within 72 hours accounted for approximately 380 encounters/month in RHTC. Revisits to ED within 72 hours accounted for almost 2-5% of all ED encounters in RHTC. These unplanned revisits to the ED from December 2021 to January 2022 showed a record of a total of 30,115 patients. Of these patients, 766 (2.5%) revisited the ED within 72 hours; out of them, 182 were excluded due to number of reasons including planned visit by emergency team of RHTC, different complaint by the patient compared to the first visit to the ED, patient refused medical care or was discharged by inpatient specialty. Thus, a total number of 584 (1.9%) patients were included in the current research as shown in Figure 1.

**Figure 2:**
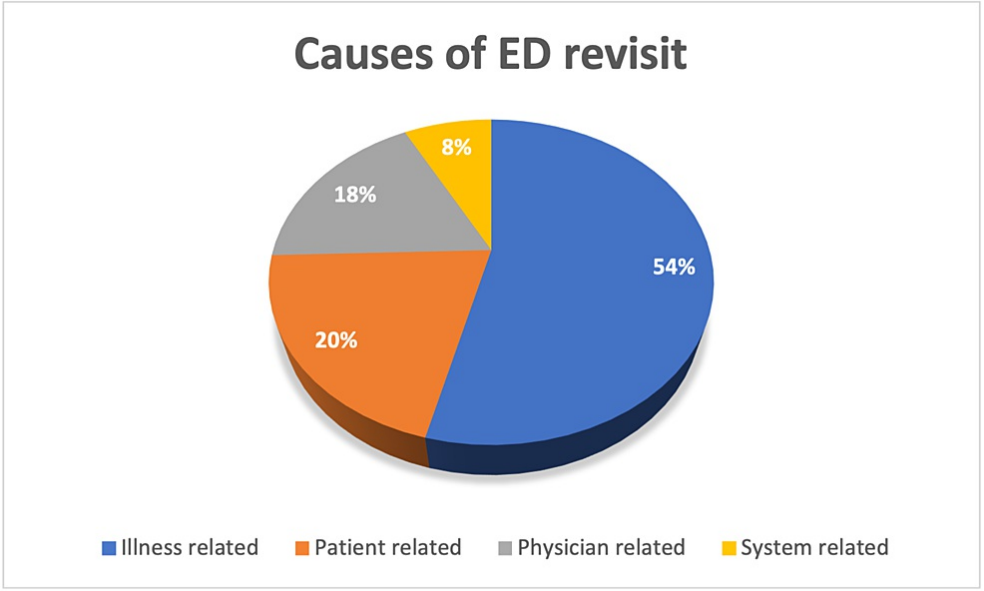
ED revisits causes.

As described in Table 2, the current study participants’ mean age was 33 years (ranging from seven months to 96 years). The majority of the study participants (63%) were males. Illness related factors accounted for 315 (54%) of the ED revisits, followed by patient related factors 120 (20%) and physician related factors 104 (18%) in addition to the system related factors which accounted for 45 (8%) (Figure 2).

**Table 2:** Demographic data of the study.

Variables		Frequency	Percentage (%)
Gender	Male	367	63
	Female	217	37
Age	<10 years	25	4
	11-18 years	28	5
	19-29 years	167	29
	30-49 years	242	41
	50-69 years	81	14
	>70 years	41	7

**Figure 3:**
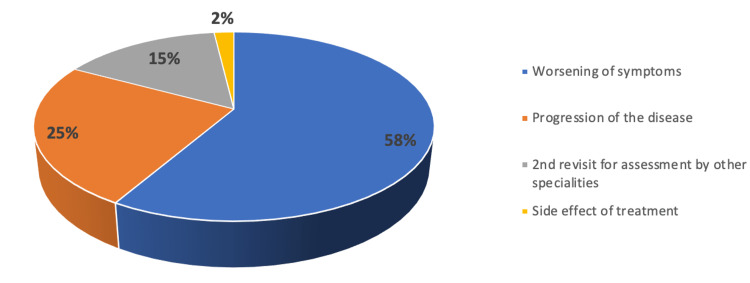
Illness-related factors.

**Figure 4:**
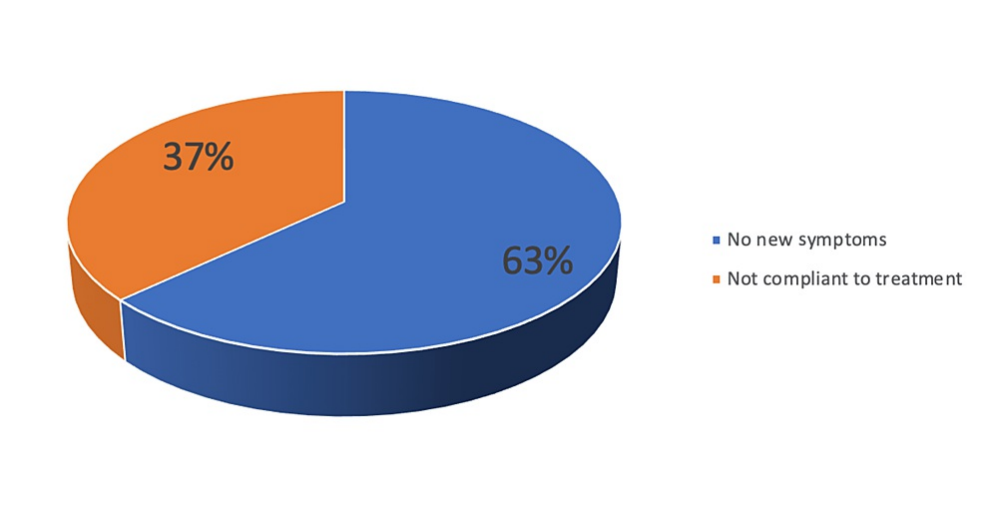
Patients-related factors.

Out of the 315 patients who reported illness-related causes, 184 (58%) came back due to worsening symptoms, 77 (25%) came back due to disease progression, while 48 patients (15%) represented to an ED within 72 hours to be seen by other specialties. Only six patients (2%) came back complaining of a side effect of the treatment provided on the first visit as shown in Figure 3. Further, major cause of ED revisits for the patient-related factor was being not compliant with the treatment given (66%) and patients’ preferences to revisit ED where no new symptoms have developed 41 (34%) (Figure 4).

**Figure 5:**
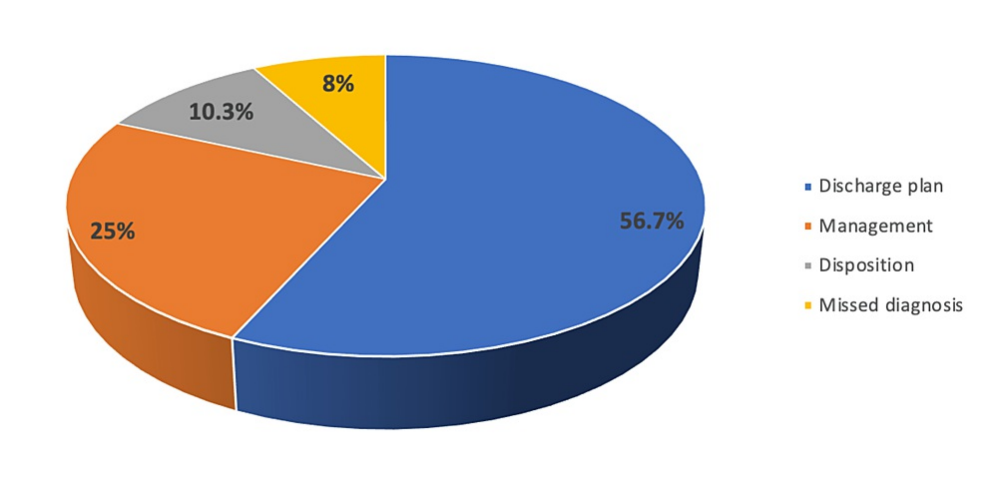
Physician-related factors.

**Figure 6:**
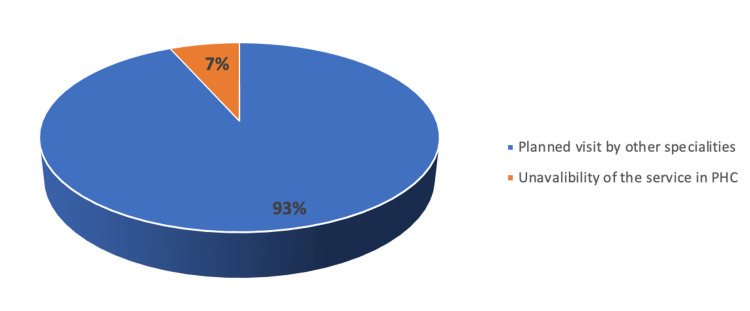
System-related factors. PHC: primary health care

Furthermore, physician-related reasons for ED revisits ranked third in the subcategories, from which the majority of the reasons were related to discharge planning, followed by emergency management, disposition, and finally missed diagnosis at 56.7%, 25%, 10.3%, 8%, respectively (Figure 5). Moreover, system-related reasons as shown in Figure 6 consisted of any planned visit to the ED organized by other medical specialties (93%) and due to unavailability of the service in the Primary health care center (7%).

**Figure 7:**
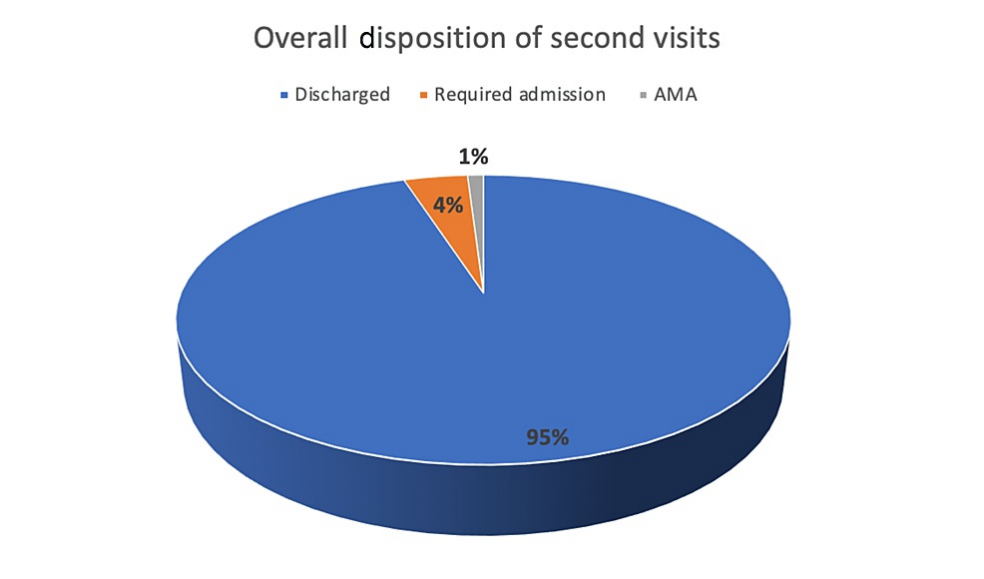
Overall disposition of the patients who revisited the ED within 72 hours. AMA: against medical advice

In the second revisit, most patients were discharged (95%), only 24 patients (4%) required admission, and six patients (1%) refused emergency care and signed against medical advice (AMA). No mortality cases in the current study population were reported as shown in Figure 7.

Discussion

According to the revisit timing of 72 hours, the unplanned ED revisit rate ranged from 0.99% to 5.8% as per the records of the previous studies [1-9], where revisit of 20% participants was mentioned in one research with the revisit duration of 30 days [12]. In the current study population, the revisit rate was 1.9% in the same range as reported by other researchers from other ED revisit within 72 hours [1-9].

In fact, it is important to highlight that electronic medical records might give higher rate of ED revisits, as all ED revisits would be captured on the electronic medical report, which needed manual filtering of the data to exclude planned ED revisit like review wounds or ankle sprain, patients attending with different complaints, and patients who were discharged by inpatient team or patient was discharged on request. This is also reported in a previous study that the development of EMR would make the available clinical information of patients at broad spectrum, inciting more comprehensive results on utilization of healthcare services by the patients and their characteristics comprehensively [13].

Additionally, illness-related reasons were the most common reason for ED revisits (58%) of the total unplanned revisit cases, similar to other studies [12]. In the current study, further subcategories of the illness-related cases were made to further understand the reasons related to ED revisits and identified that 58% of the illness-related cases were secondary to worsening of the patient clinical condition, mainly worsening of pain symptoms (35%). For example, patient with renal colic was managed and discharged with analgesia for follow-up, but the patient revisited ED with worsened pain. Further, patient with non-traumatic back pain also revisited ED with pain worsening complaint. Other cases of clinical deterioration include patients with gastroenteritis who revisited ED and had recurrent vomiting, and a patient with upper respiratory tract infection who developed lower respiratory tract infection, provided that the patient care and management in the initial visit were optimized as per the guideline.

The second common cause of unplanned revisits to the ED in the current study was patient related (20%). For example, a case of a patient who self-removed his back slab POP which was applied for ankle trauma. Similarly, a previous study conducted in Taiwan also found that the second most common cause for ED revisits was patient-related factors but, accounted for 10.9%, which is less than the result of this study [8].

Physician-related ED revisits accounted for 18% of the total unplanned revisits, which is similar to the findings of the other studies which showed 16.7% of the ED revisits due to physician-related causes [2]. Discharge planning was the commonest cause (56.7%) for physician-related revisit; examples would be inadequate oral analgesia upon patient discharge or another example would be no specialist outpatient appointment was requested for the patient followed by medical management (25%) examples include insufficient medical assessment in a patient with traumatic shoulder dislocation, in which no assessment for injuries in other parts of the body and a missed chest injury with rib fracture was identified on the second visit to ED. In another case in an elderly patient with acute urine retention, urine catheter was inserted and then removed prior to discharge, he revisited again with acute urine retention. The third reason was inappropriate disposition which represented 10.6% of the physician-related causes. For example, an elderly patient with community-acquired pneumonia with high CURB-65 score was discharged home and revisited for escalation of his treatment (the need for intravenous antibiotic and oxygen support). The fourth reason was categorized as a missed diagnosis which represented 8% of the physician-related causes. For example, a patient presented with facial swelling diagnosed with allergy and patient was found to have ACE inhibitors angioedema or a patient with right abdominal pain misdiagnosed with renal colic and was found to have appendicitis.

Last reason related to unplanned ED revisit was system related (8%), which is an important factor to be highlighted where doctors from other specialties in the hospital request their patients to continue their care in the ED due to the unavailability of the medical care in the health care system. For example, patients with multiple sclerosis were asked to get their methylprednisolone infusion in the ED due to the lack of service during the weekends and public holidays.

Based on our findings in our study we strongly recommend educating the emergency physicians about the importance of discharge planning for emergency patients, in addition to educating the patients about the emergency health services, hoping this will reduce the number of revisits to the emergency department.

Limitations

Every study had some limitations. One of the limitations of this study was its retrospective nature which relied on the emergency physician documentation in the electronic medical record system of hospital and that was insufficient to provide clear explanation for the patient revisit in some cases which required the research team to do further workup to identify the cause for the patient revisit. In addition, this research only included the government hospital in the state of Dubai and the private hospitals were not included due to the lack of access to the electronic medical records of the private hospitals, thereby patients that represented to ED within 72 hours to a private facility might have also been missed.

## Conclusions

The most accountable reason for the unplanned revisits to the ED within 72 hours was illness related to the progression of the disease as the most common cause for the revisit. And the second cause was patient-related factors, and the third cause was physician-related reasons which accounted for only 18% of the total number of revisits with discharge planning as the highest cause identified. Only 5% of the patients who revisited the ED identified a need for hospital admission and no mortality was identified in the patients who revisited the ED in this retrospective research.

However, this is the first study in the UAE that looked into such an important KPI for acute medical care. Further studies are required to look into this matter with possible further analysis of the reasons behind the patient-related revisits to ED like accessibility to health care, and financial and communication barriers in the near future.
